# Lysyl-Phosphatidylglycerol: A Lipid Involved in the Resistance of *Staphylococcus aureus* to Antimicrobial Peptide Activity

**DOI:** 10.3390/antibiotics14040349

**Published:** 2025-03-28

**Authors:** Andrea Vásquez, Chad Leidy, Marcela Manrique-Moreno

**Affiliations:** 1 Chemistry Institute, Faculty of Exact and Natural Sciences, University of Antioquia, A.A. 1226, Medellin 050010, Colombia; andrea.vasquezz@udea.edu.co; 2 Biophysics Group, Physics Department, Universidad de los Andes, Bogotá 111711, Colombia

**Keywords:** lysyl-PG, *Staphylococcus aureus*, lipid–peptide interaction, bacteria resistance-mechanism

## Abstract

Lysyl-phosphatidylglycerol (lysyl-PG) is one of the major lipids found in bacterial membranes; it is synthesized by attaching lysine to the headgroup of phosphatidylglycerol. First identified in *Staphylococcus aureus* in 1964, lysyl-PG is now recognized as a virulence factor that protects *Staphylococcus aureus* from antimicrobial agents, such as cationic antimicrobial peptides and phospholipase A2 type IIA. Under normal growth conditions, *Staphylococcus aureus* membranes are negatively charged due to a high proportion of anionic lipids, such as phosphatidylglycerol and cardiolipin. This intrinsic anionic charge helps attract positively charged antimicrobial agents to the membrane surface, increasing their disruptive activity. The presence of lysyl-PG reduces electrostatic interactions, making the membrane less susceptible to cationic agents. The biosynthesis of lysyl-PG is mediated by the multiple peptide resistance factor (MprF) enzyme, which catalyzes the modification of phosphatidylglycerol and translocation of lysyl-PG to the outer membrane in the presence of antimicrobial agents. However, several studies indicate that lysyl-PG not only responds to the presence of antimicrobial agents but can fluctuate based on environmental factors such as oxygen availability and nutrient composition. Acidic conditions and nutrient-rich media often result in increased lysyl-PG production, suggesting that bacterial membranes can be resistant to cationic antimicrobial agents even in their native state. Recent studies propose that targeting MprF to inhibit lysyl-PG biosynthesis could be a promising strategy to counter antimicrobial resistance. This review highlights the role of lysyl-PG in modulating membrane charge and its influence on antimicrobial agent efficacy and discusses a possible strategy for treatment by targeting lysyl-PG synthesis.

## 1. Introduction

*Staphylococcus aureus* (*S. aureus*) is one of the most frequent worldwide pathogenic bacteria responsible for severe infections in humans and is well known for its adaptive acquisition of antibiotic resistance, which has led to the emergence of different strains that show selective tolerance to nearly all clinically available antibiotics [1]. The 2022 Global Antimicrobial Resistance and Use Surveillance System (GLASS) report highlighted the alarming detection of resistant *S. aureus* isolates collected from hospitals with both inpatient services and outpatient clinics, with median rates of methicillin-resistant *S. aureus* of 35% in at least 76 countries [2]. Furthermore, in 2023, Hasanpour et al. found widespread methicillin-resistant *S. aureus* colonization among elderly care residents worldwide, with increased risk factors, including prior methicillin-resistant *S. aureus* infections, hospitalizations, antibiotic use, diabetes, and medical device use [3].

Different resistance mechanisms in *S. aureus* play a crucial role in its survival and persistence during antibiotic treatment [4]. A defining feature of *S. aureus* is its ability to alter its membrane composition in response to external stresses, such as antibiotic exposure [5,6]. Changes in lipid bilayer composition, including variations in lipid type and charge, can influence membrane characteristics such as fluidity, permeability, membrane potential, and thickness, which, in turn, affect antibiotic penetration and efficacy [7,8]. The ability of the bacterium to modify its membrane structure not only aids in resistance but also influences virulence factor expression, allowing it to evade immune defenses [9,10]. The interplay between membrane adaptation and virulence factor expression makes *S. aureus* a formidable pathogen, complicating treatment strategies and enabling persistent infections in the host.

In the literature, several virulence factors have been described in *S. aureus*, including the presence of alpha toxin, adhesins, protein A, coagulase, and enterotoxins [10]. Alpha toxin, a pore-forming cytolysin, plays a critical role in disrupting host cell membranes, leading to cell lysis and tissue damage [11]. Adhesins, such as fibronectin-binding proteins (FnBPs) and clumping factors (ClfA and ClfB), facilitate bacterial attachment to host tissues, promoting colonization and infection [12]. Protein A is a surface protein that binds to the Fc region of immunoglobulins, interfering with opsonization and phagocytosis, thereby aiding immune evasion [13]. Additionally, the polysaccharide capsule and the biofilm-forming ability of the bacterium favor immune evasion, while the presence of specific enzymes degrades or modifies antibiotics, enhancing the possibility of survival [14]. A well-established virulence factor in *S. aureus* is the multiple peptide resistance factor (MprF), an enzyme that produces lysyl-PG, a positively modified phospholipid. MprF catalyzes the transfer of lysine from tRNA to phosphatidylglycerol (PG), forming lysyl-PG, which then translocates to the outer membrane leaflet [10,15,16,17]. This modification is crucial as it alters the membrane surface charge. The two main lipids that constitute the *S. aureus* membrane are PG and cardiolipin (CL), which are both anionic due to the presence of single-charged anionic phosphates in the headgroup: one phosphate group in the case of PG and two phosphate groups in the case of CL. The addition of lysyl to the headgroup of PG lipids switches the net charge of the phospholipid by adding two positive charges to the headgroup. For this reason, it has been proposed that the synthesis of cationic lysyl-PG has the main purpose of altering the membrane surface charge of the bacterium, with consequences in the interactions with cationic antimicrobial peptides (cAMPs). It is suggested that the inclusion of this positively charged lipid decreases the overall negative charge of membranes, thus reducing the affinity of positively charged molecules towards the bacterial membrane, therefore protecting the membrane from the lytic effects of cationic antimicrobial agents [15,18].

The protective effect of lysyl-PG may not be restricted to reducing the affinity of antimicrobial peptides to the membrane surface. Kilelee et al. show that the presence of lysyl-PG does not significantly alter the binding affinity of a synthetic antimicrobial peptide (6W-RP-1) to the membrane. Instead, peptide-induced leakage was drastically reduced in the presence of lysyl-PG. These results suggest that lysyl-PG plays a structural role in the membrane, preventing peptide insertion, most likely by inducing membrane condensation and therefore increasing the energetic cost of peptide insertion [19].

The biosynthesis of lysyl-PG occurs in the luminal face of the membrane. The dual-domain structure of MprF facilitates both lysyl-PG synthesis and translocation towards the outer monolayer, which is essential for infection persistence. According to the study by Ernst et al., these domains work together to reduce the net negative charge of the outer leaflet of the bacterial membrane. Mutations disrupting either the lysyl-PG synthesis or translocation domain led to a significant decrease in resistance to cAMPs, underscoring the necessity of both functions for effective immune evasion. Interestingly, this study also demonstrated that while these protein domains are functionally complementary, they do not need to be covalently linked to perform their roles effectively [20]. MprF-mediated charge modulation has become a focal point in the study of bacterial resistance mechanisms, particularly in Gram-positive pathogens such as *S. aureus*. This review traces the study and characterization of lysyl-PG and its relationship with antimicrobial resistance in *S. aureus* and other bacteria and discusses the influence of the modified lipid on membrane charge and antimicrobial peptide and antibiotic interactions. The objective of this study was to provide a global understanding of how lysyl-PG biosynthesis contributes to membrane resistance and to outline its potential as a target for antimicrobial therapeutics.

## 2. *Staphylococcus aureus*

*Staphylococcus* is a genus of Gram-positive bacteria and is typically spherical and arranged in irregular clusters. These bacteria are aerobic facultative, grow easily on a variety of media, and can ferment carbohydrates, producing pigments from white to deep yellow [21,22]. While *S. aureus* is a normal inhabitant of human skin and mucous membranes, it can also act as a pathogen, causing infections that range from superficial skin lesions to severe sepsis [23,24]. Pathogenic strains such as *S. aureus* are notable for their hemolytic activity and plasma coagulation and release various extracellular enzymes and toxins that help them evade the host immune response [10]. The *Staphylococcus* genus includes over 45 species, with more than a dozen known to colonize humans. The most clinically significant species are *S. aureus*, *S. epidermidis*, *S. lugdunensis*, and *S. saprophyticus* [22]. *S. aureus* is classified as coagulase-positive due to its production of coagulase, an enzyme that converts fibrinogen to fibrin, enabling blood clot formation. This fibrin coating helps the bacterium evade immune defenses by avoiding phagocytosis [25,26]. In addition, *S. aureus* is primarily a commensal organism, colonizing 20 to 40% of asymptomatic individuals, especially on mucous membranes and skin [27,28,29]. The transmission of *S. aureus* occurs mainly through direct human-to-human contact, though zoonotic transmission to animals has also been reported [30]. Infections range from skin conditions to more serious illnesses such as bacteremia, endocarditis, pneumonia, and foodborne illnesses, making it a significant health concern [10,23].

The cell wall and membrane are fundamental structures in bacteria, essential for maintaining cellular integrity, shape, and function. Understanding these components is crucial for comprehending bacterial physiology and the mechanisms of antibiotic action. For instance, Strominger et al. investigated the cell wall of *S. aureus*, finding that it was composed of amino acids, carbohydrates, and peptides. They made use of ion exchange chromatography, which was crucial to separate and quantify these cell wall components. Furthermore, they identified a strong correlation between cell wall composition and penicillin action, observing that penicillin disrupts the biosynthesis of cell wall precursors. This quantitative chromatographic analysis allowed them to establish a direct correlation between cell wall composition and the biosynthetic pathways affected by penicillin [31].

**Figure 1:**
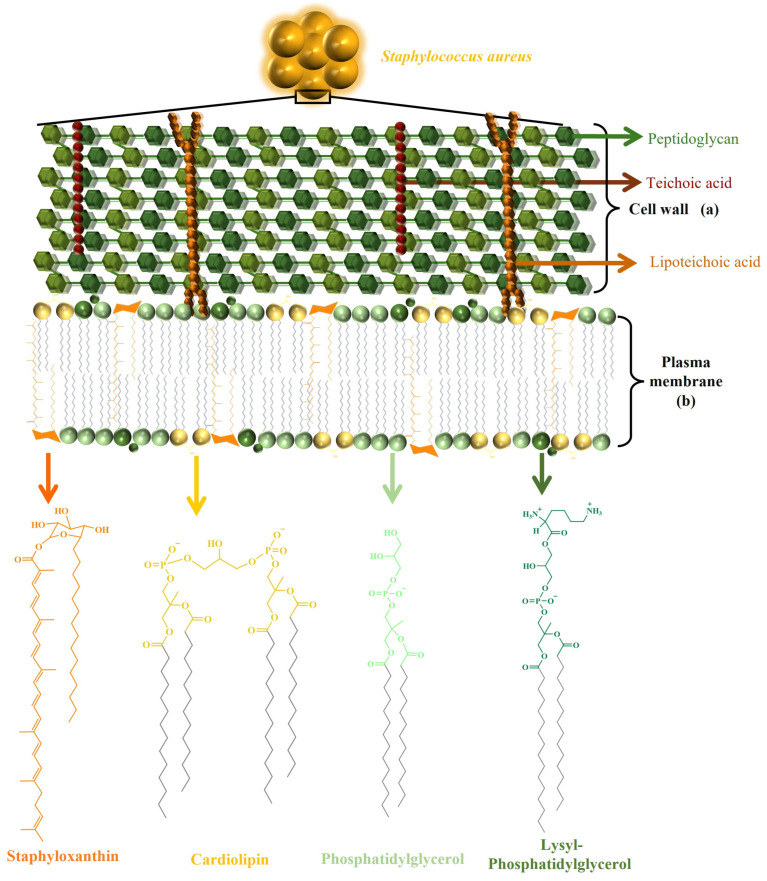
Structural model of *S. aureus* membrane. (**a**) Representation of the cell wall, including peptidoglycan, teichoic acid, lipoteichoic acid, and (**b**) the plasma membrane, illustrating the distribution of key phospholipids, such as PG, lysyl-PG, and CL, along with the carotenoid pigment STX and their structures.

The wall and cell membrane of *S. aureus* (Figure 1) is built of several components. The primary component, peptidoglycan, forms a thick layer, approximately 20 to 40 nm in thickness, surrounding the cytoplasmic membrane. This layer consists of glycan chains made of alternating N-acetylmuramic acid and N-acetylglucosamine residues, cross-linked by short peptide chains [32]. The typical stem peptide structure includes L-alanine, D-iso-glutamine, and L-lysine and is connected with a pentaglycine bridge to the terminal D-alanine residue. In addition to peptidoglycan, the cell wall contains teichoic and lipoteichoic acids, both of which are phosphate-rich glycopolymers. Wall teichoic acids are covalently linked to peptidoglycan, while lipoteichoic acids are anchored in the cytoplasmic membrane (Figure 1a). These components are crucial not only for maintaining cell wall integrity but also for mediating interactions with the host immune system [17,33]. Additionally, modifications to teichoic acid, such as D-alanylation catalyzed by the DltABCD system, reduce the negative charge on the cell surface, which repels cAMPs and enhances bacterial survival during infections. This charge modification also promotes adherence to host cells, aiding colonization and infection [34,35].

## 3. Membrane Composition

The cell membrane is a complex biological structure that separates the cytosol from the extracellular environment while maintaining selective permeability [36]. In *S. aureus*, the cytoplasmic membrane functions as the primary interface between the cell and its surroundings. Alterations in the composition of bacterial cell membranes have been shown to influence their adaptability and pathogenicity [32]. The main lipid component of the *S. aureus* membrane is PG (Figure 1b), which constitutes 58% to 80% of the phospholipids present in the bacterium [37,38]. PG plays a vital role in maintaining membrane stability due to its anionic nature, primarily stemming from the negatively charged phosphate group. This charge influences membrane dynamics, including its ability to undergo cooperative phase transitions that depend on ionic strength. As Epand and Hui note, PG maintains stable phase transitions and structural integrity in the presence of salts like NaCl, ensuring the preservation of the bilayer architecture. At low ionic strength, however, these phase transitions become more pronounced, destabilizing the membrane. However, under physiological conditions, salt concentrations are sufficient to stabilize PG content lipid membranes [39]. Additionally, PG’s capacity to form multilamellar structures under high ionic conditions contributes to maintaining membrane morphology, whereas low ionic strength leads to notable structural changes, such as the formation of smaller particles or half-shells. These findings emphasize how PG’s negative charge and its response to ionic environments are critical for preserving membrane stability and functionality under physiological conditions [39]. Structurally, PG exhibits an amphipathic nature, with its hydrophilic headgroup and hydrophobic tail region interacting to influence its packing behavior in the membrane, which can contribute to the overall shape and organization of the lipid bilayer [40].

Another main component of the membrane is cardiolipin typically constitutes 4 to 20% of the *S. aureus* membranes [38,41]. CL consists of four fatty acids and is synthesized in prokaryotes through a transphosphatidylation reaction catalyzed by the cardiolipin synthases Cls1 and Cls2. These enzymes facilitate the fusion of two PG molecules to form CL, releasing glycerol in the process as a byproduct [42]. Under normal growth conditions, Cls2 predominantly mediates CL synthesis, while under acidic stress, Cls1 becomes essential for its production [43]. In *S. aureus*, CL concentration increases during the transition from the exponential to the stationary growth phase, while PG simultaneously decreases [44]. Also, the anionic nature of CL allows it to interact with membrane proteins that often comprise positively charged amino acid residues; this interaction stabilizes membrane proteins and contributes to the overall stability of the membrane structure. For instance, Yeo et al. demonstrated that cardiolipin directly interacts with sensor kinases such as SaeS, which are critical for regulating virulence factors in *S. aureus*. This binding enhances the activity of these proteins, ensuring proper cellular responses to environmental challenges, demonstrating that CL is also required for bacterial virulence [45].

The dynamic composition of the *S. aureus* plasma membrane, particularly the abundance of PG and CL, enables the bacterium to adapt to various stress factors, such as pH changes, temperature fluctuations, and exposure to antimicrobial agents, by altering its membrane proteome and sensory regulation pathways [8]. Moreover, *S. aureus* has evolved mechanisms to modify its membrane composition, thereby increasing its pathogenic potential. One such mechanism involves the MprF enzyme, which synthesizes lysyl-PG by adding lysine to PG.

In addition to phospholipids, other membrane components, such as staphyloxanthin (STX), a carotenoid pigment, provide *S. aureus* with protection against oxidative stress and contribute to its characteristic golden color. STX plays a crucial role as a virulence factor, enabling the bacterium to evade the host immune response. Specifically, STX acts as an antioxidant against reactive oxygen species produced by the immune system, a function attributed to its unique chemical structure, which features multiple conjugated double bonds [46]. The main STX species have acyl chains of 15, 17, and 19 carbons [47,48]. Studies suggest that STX constitutes 2–23 mol% of the total carotenoid population [49]. Additionally, membrane-associated components, such as staphyloxanthin in *S. aureus*, enhance bacterial survival against host immune responses by promoting oxidative stress resistance, illustrating the complex role of membrane features in the bacterium’s pathogenicity [50]. A deeper understanding of the lipid composition and adaptability of *S. aureus* membranes not only provides insights into its survival strategies but also enables the identification of potential targets for developing therapeutic strategies to fight their resistance to antimicrobial agents.

## 4. Aminoacyl Lipids: From Discovery to Function

In 1959, researchers using chromatographic techniques and radioactive labeling quantified amino phospholipids in different membranes, marking a significant advance in the understanding of membrane lipid composition. These studies identified amino phospholipids in microorganisms, such as *Penicillium chrysogenum* and *Bacillus megaterium*; ascites tumor cells; and liver cells [51,52,53], even though the function and the structures of these phospholipids were still poorly understood. In 1962, Dr. Macfarlane made a significant discovery in *S. aureus* by identifying a lipid component bound to a nitrogenous compound that accounted for 66% of the total phospholipids. Chemical tests for inositol, glucosamine, and N-acetylglucosamine were negative, suggesting a unique amino acid association [54]. Further research revealed that *S. aureus* produces only one aminoacyl lipid, lysyl-PG. Dr. Macfarlane made another significant discovery with *Clostridium welchii*, identifying lipoamino acids such as O-amino acid esters of phosphatidylglycerol. Through acidification extraction, infrared analysis, and 2,4-dinitrophenylhydrazine testing, he proposed that the amino acid was attached to the α1 carbon of the unacylated glycerol [55].

Building on these findings, Houtsmuller and Van Deenen, in 1963, identified a minor phospholipid in *Bacillus cereus* as an O-ornithine ester of PG. They purified the compound and confirmed that ornithine was linked to PG via an ester bond [56]. A year later, the same researchers quantified the phospholipid composition of *S. aureus* and observed that adding glucose to the growth medium significantly increased the lysyl-PG content. Houtsmuller and van Deenen attributed this to the drop in pH resulting from glucose fermentation, as the acidic environment favored lysyl-PG production [57], a finding corroborated by subsequent studies [37,58,59]. Interestingly, when the pH was restored, lysyl-PG levels decreased while PG levels increased, highlighting the dynamic nature of bacterial phospholipid metabolism and the significant influence of environmental conditions, such as nutrient availability and pH, on *S. aureus* membrane composition [57]. Later, in 1965, Houtsmuller and van Deenen continued their research into lysyl-PG, studying how pH affects the phospholipid composition of *S. aureus*. They successfully isolated a pure amino acid derivative of PG, identifying it as an L-lysine ester of 1,2-diacyl-glycero-3-phosphorylglycerol. They concluded that a lysine molecule is esterified to a PG molecule, with both amino groups of lysine remaining free [60].

Building on this finding, Lennarz and coworkers demonstrated that cell-free extract from *S. aureus* is capable of catalyzing the incorporation of C14-labeled L-lysine into lysyl-PG. This significant discovery highlights the enzymatic activity present in these extracts and its role in lipid biosynthesis. They proposed that this reaction involves at least two enzymatic steps: first, lysine is activated by forming lysyl-sRNA, followed by a particulate enzyme that transfers the lysyl group from lysyl-sRNA to PG. This work represented a major advance in understanding the biochemical pathways of bacterial lipid synthesis [61]. Expanding this knowledge, Gould and Lennarz discovered that the enzymatic synthesis of lysyl-PG from lysyl-sRNA occurs in a variety of microorganisms. They also found that some organisms can enzymatically produce at least two distinct aminoacyl derivatives of PG from the respective aminoacyl-sRNA derivatives [62].

Later, Gould and Lennarz examined the *S. aureus* phospholipid content under different environmental conditions. They found that at pH 7.0, the lysyl-PG to PG ratio was low, whereas at an acidic pH of 5.2, PG levels decreased, leading to a relative increase in lysyl-PG. This suggests that lysyl-PG remains stable even when PG synthesis is inhibited. Furthermore, analysis of lysyl-PG turnover showed that upon decomposition, lysine is released as free lysine rather than being used for protein or cell wall synthesis. Notably, they observed that erythromycin and cephalothin do not affect the breakdown of lysyl-PG, even though they inhibit other important cellular processes like protein synthesis and cell wall formation. This suggests that lysyl-PG metabolism operates independently of the pathways affected by these antibiotics [59]. Based on this fundamental knowledge, Nahaie and coworkers extended their studies to other *Staphylococcus* species. The researchers identified PG, lysyl-PG, and diphosphatidylglycerol (cardiolipin) in 74 strains across 13 species. They observed that coagulase-negative strains typically contained only trace amounts of lysyl-PG, suggesting variability in lipid composition within the genus [63].

For nearly four decades, the biological function of aminoacyl-PG remained unclear. However, while screening *Staphylococcus* mutants for antibacterial peptide sensitivity, Peschel et al. identified *mprF*, a gene essential for resistance to host defense peptides like defensins and protegrins. Their analysis showed that *mprF* mutants were unable to modify PG with L-lysine, a modification that reduces membrane surface negativity and likely repels cationic peptides. As a result, *mprF* inactivation led to increased binding of antimicrobial peptide. They also observed that human neutrophils cleared the *mprF* mutant strain more quickly, and it exhibited reduced virulence in mice, underscoring the importance of defensin resistance in *S. aureus* pathogenicity. Additionally, they found that *mprF* shared no homology with known genes at the time, though related genes were later identified in pathogens such as *Mycobacterium tuberculosis*, *Pseudomonas aeruginosa*, and *Enterococcus faecalis*. As a result, MprF is now recognized as a key virulence factor and a promising target for combating multidrug-resistant bacteria [15].

The discovery and characterization of lysyl-PG in *S. aureus* have significantly deepened our understanding of bacterial membrane composition, highlighting its critical role in antibiotic resistance and host–pathogen interactions. Identified as a key factor in *S. aureus* resistance to cAMPs, lysyl-PG modulates membrane charge, thereby enhancing bacterial survival in hostile environments. However, research has shown that lysyl-PG, along with other aminoacyl phospholipids, is not exclusive to *S. aureus* or previously studied organisms. This lipid is found across Gram-positive and some Gram-negative bacteria, where it similarly contributes to membrane stability and resistance [64,65]. This suggests an evolutionarily conserved function in bacterial defense, positioning lysyl-PG as a promising target for the development of novel antimicrobial therapies. The following section reviews lysyl-PG across various bacterial species, providing insights on the broad significance of this lipid modification.

## 5. Adaptive Membrane Lipid Modifications in Bacteria: Role of Lysyl-PG and Related Lipoamino Acids

Bacteria inhabit diverse environments, where they face fluctuations in salinity, temperature, pH, and exposure to antimicrobial agents. To survive, bacteria adjust their membrane composition, structure, and properties during growth and in response to external stress. This membrane adaptation is crucial for survival as bacterial membranes are primary sites of interaction with host defenses, antimicrobial peptides, and antibiotics. For instance, some bacteria modify their membrane lipids to enhance survival, reducing the negative charge of the membrane by adding amino acids such as lysine and alanine to PG and CL to reduce the susceptibility to antimicrobial cationic peptides and adjusting lipid composition to maintain membrane fluidity and integrity under varying environmental conditions [5,15,18,66].

The addition of lysine alters the net charge of PG from −1 to +1 and CL from −2 to 0, creating cationic or zwitterionic molecules that lower the overall negative surface charge [67]. These aminoacylated lipids are often called lipoamino acids, although it may be more precise to refer to them as complex lipoamino acids. This distinction helps differentiate them from simpler forms, such as ornithine lipids, which consist of a fatty acid linked to an amino acid and are thought to serve similar functions [56,68]. Furthermore, complex lipoamino acids derived from CL have been identified in specific bacterial species. For example, Fischer and Leopold (1999) reported the discovery of lysyl-CL, a novel form of aminoacylated CL previously unknown in nature, as the most abundant aminoacylphospholipid in four species of Listeria. Their study revealed that *Listeria innocua* contained 12% lysyl-CL, *Listeria monocytogenes* 3% to 12.3%, *Listeria seeligeri* 37.4%, and *Listeria welshimeri* a notably high level of 47.3%, all during the stationary phase [69].

Lysyl-PG has been detected in the membranes of at least 43 Gram-positive and Gram-negative bacterial species (Table 1). Since its discovery, numerous studies have focused on examining the structure of lysyl-PG and its role in generating a positive surface charge that inhibits the activity of cAMPs. Initially identified in *Staphylococci* and later found in *Bacillus*, *Pseudomonas*, *Listeria*, *Mycobacteria*, and *enterobacteria*, lysyl-PG has emerged as a key amino phospholipid in several species [54,69,70,71,72,73]. It serves as a resistance mechanism by reducing PG levels and decreasing overall surface negativity. Some researchers have suggested that this modification leads to an asymmetric lipid distribution across the membrane leaflets, lowering the binding affinity of cationic antimicrobial agents [74,75]. This modification is made possible by the activity of aminoacylphosphatidylglycerol synthases (aaPGSs), enzymes that catalyze the transfer of amino acids from aminoacyl-tRNA to the phospholipid PG or CL in bacterial membranes. These enzymes exhibit distinct amino acid specificities, with each enzyme typically being specific to a particular amino acid, enabling them to modify phospholipids in response to environmental conditions, such as nutrient variations during infection [76,77]. For instance, some aaPGSs, like the one from *S. aureus*, are highly specific to their substrates, while others, such as the enzyme in *Enterococcus faecium*, can recognize multiple amino acids [77]. A notable example is aaPGSs from *Anoxybacillus rupiensis*, which can modify PG with alanine, arginine, lysine, and also lysinate CL [78].

This enzymatic diversity represents an evolutionary adaptation that allows bacteria to modify their membranes in response to ecological or host-specific pressures. Although this lipid has become a key component of bacterial resistance, it is considered that bacteria can reverse this modification through succinylation reactions. In 2016, Atila et al. described the N-succinylation of L-lysyl-PG in *Bacillus subtilis*, demonstrating that N-succinyl-lysyl-PG enables dynamic charge reversal, shifting from −1 to +1 and back to −1. This charge modulation likely aids bacterial adaptation by altering the membrane’s electrostatic properties as needed, affecting interactions with antimicrobial agents and maintaining membrane stability. The N-succinylation process involves an amide bond between succinic acid and lysine, generating a charge change that stabilizes membrane lipids. This suggests broader regulatory roles for lipids in bacterial physiology and gene expression [87]. This adaptive mechanism extends beyond *Bacillus subtilis*; in 2018, researchers detected N-succinyl-lysyl-PG in *Staphylococcus haemolyticus*, suggesting that its presence may result from mobile genetic elements across *Staphylococcus* species, further supporting its role in bacterial adaptation to host defense [100].

## 6. Mechanisms of Lysyl-PG Biosynthesis and Translocation: The Role of MprF

**Figure 2:**
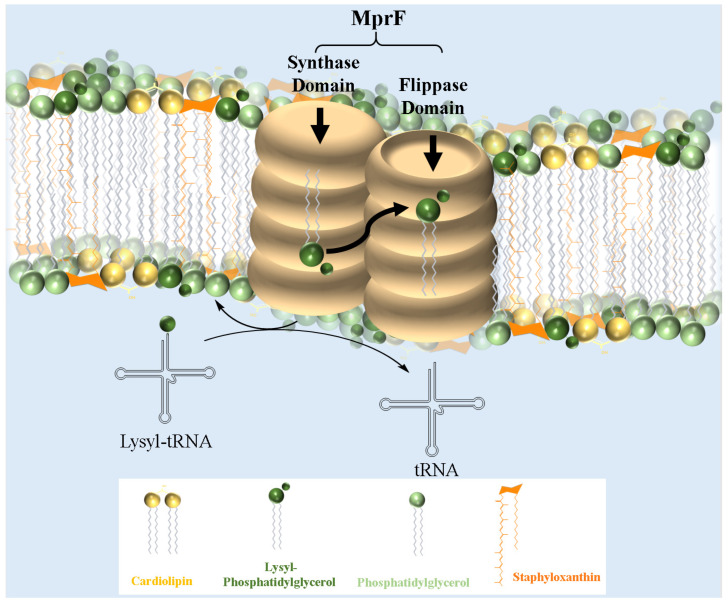
Schematic representation of *S. aureus* membrane model with lysyl-PG synthesis and MprF localization.

MprF, a crucial virulence factor in *S. aureus*, plays a key role in resistance and directly contributes to pathogenicity. This bifunctional enzyme operates via two distinct domains: the synthetase domain, which catalyzes the addition of lysine to PG to produce lysyl-PG, and the flippase domain, which translocates lysyl-PG to the outer membrane monolayer [20,73]. First, in the synthetase domain, the biosynthesis of lysyl-PG depends on aaPGSs, which transfers amino acids from aminoacyl-tRNAs to the polar headgroup of PG (Figure 2). Once synthesized, the lysyl-PG is then translocated to the outer membrane through the flippase domain, completing the modification process. The flippase domain is equally critical, as it fully activates lysyl-PG’s resistance function by translocating it to the outer membrane monolayer. Located at the N-terminus of MprF, this domain consists of multiple transmembrane segments that transport lysyl-PG from the inner to the outer membrane monolayer. Although the precise molecular mechanism is still under investigation, it is believed to rely on an energy-dependent process, possibly driven by the proton motive force, since this domain lacks ATP-binding motifs [75]. Mutations in this domain, especially those linked to daptomycin resistance, highlight its role in cAMP resistance and MprF-mediated virulence [103]. Slavetinsky et al. (2012) further demonstrated that MprF flippases can efficiently translocate both lysyl-PG and alanyl-PG. However, the synthesis and translocation of lysyl-PG conferred a significantly higher level of resistance to cationic antimicrobial peptides (cAMPs) and daptomycin compared to alanyl-PG. This finding highlights the critical role of MprF not only in modifying the bacterial membrane composition but also as an essential virulence factor in *Staphylococcus aureus*. This study also reveals that the flippase domains of MprF exhibit broad substrate specificity, enabling the efficient translocation of both lipid forms. Understanding this substrate specificity could aid in developing targeted inhibitors that disrupt MprF’s flippase activity, potentially compromising multiple resistance mechanisms simultaneously. Furthermore, it underscores the importance of considering the overall lipid composition of the membrane, which plays a key role in influencing antimicrobial resistance [104].

Through the redistribution of lysyl-PG, MprF effectively modulates membrane charge to counteract cAMPs and preserve membrane integrity under hostile conditions [20,77]. The evolutionary significance of MprF is evident in its nearly 350 homologs across various bacterial genera and three archaeal species within the *Methanosarcina* genus [76]. While these homologs display structural variability, they likely share a basic framework, typically consisting of a membrane-inserted amino terminus and a hydrophilic carboxyl terminus, though with differences in domain size and features among aaPGS proteins. In many bacterial genomes, especially Gram-positive ones, multiple aaPGS paralogs enhance the functional diversity within a single organism, enabling finely tuned membrane properties [77].

This diversity broadens resistance against several antimicrobial agents and environmental challenges, supporting bacterial adaptability and survival. During infection, aaPGSs play a vital role in the virulence of several pathogenic bacteria, helping them evade antibiotic effects. By modifying membrane characteristics, these enzymes strengthen resistance to a range of antibiotics and environmental stresses, making them key contributors to bacterial pathogenicity [76,77].

## 7. Role of Lysyl-PG in Modulating *S. aureus* Resistance to Antimicrobial Agents

As outlined earlier, lysyl-PG plays a crucial role in cAMP resistance in *S. aureus.* By converting PG to lysyl-PG, the bacteria alter the membrane’s net charge from negative to slightly positive, enabling the microorganism to reduce the interaction with cAMPs. Table 2 summarizes different studies that used synthetic lipids or lipid extracts to model these interactions. These studies commonly employ liposomes, supported, or monolayer models with varying proportions of lysyl-PG and PG, occasionally incorporating phosphatidylcholine (PC), phosphatidylethanolamine (PE), and glycolipids (GLs). Consistently, the findings demonstrate that lysyl-PG significantly reduces membrane susceptibility to cationic peptides, emphasizing its critical role in resistance mechanisms.

First, Peschel et al. demonstrated that a high content of lysyl-PG (above 38%) reduced the electrostatic attraction and binding of cAMPs and defensins, thereby enhancing *S. aureus* resistance. Their research also showed that inactivating the *mprF* gene, which encodes lysyl-PG synthase, increases *S. aureus* susceptibility to immune defenses. In NMRI mice, *mprF* mutants exhibited significantly lower mortality rates compared to the wild-type strains; 8 out of 24 mice infected with the wild-type strain succumbed, while no mortality occurred in mice infected with *mprF* mutants [15]. These results highlight the critical role of membrane lipid modification as a resistance mechanism in *S. aureus* pathogenicity and virulence.

Koprivnjak et al. investigated the role of charge properties in determining susceptibility to phospholipase A2 (PLA2), an enzyme known to disrupt bacterial membranes by hydrolyzing phospholipids (Koprivnjak et al., 2002). Their study focused on the interaction of PLA2 with membrane models of different *S. aureus* strains, emphasizing the potential role of lysyl-PG. To explore this, the authors analyzed two mutants: the *dltA^−^* mutant, which cannot modify teichoic acids with D-alanine, and the *mprF^−^* mutant, which lacks lysyl-PG synthesis. Both mutants exhibited increased sensitivity to PLA2, with the *dltA^−^* mutant being between 30 and 100 times more sensitive than the parent strain, and the *mprF^−^* was three times more susceptible (≤3-fold). Interestingly, the rate of the phospholipid degradation by PLA2 remained consistent across all strains tested. The authors suggest that the modest effect on sensitivity may not result from changes in membrane properties alone but rather from other bacterial alterations linked to the *mprF^−^* modification, which have yet to be identified [105].

Further research identified two additional genetic factors, *fmtC* and *lysC*, that play a role in lysyl-PG and lysine synthesis, respectively. Mutant strains with inactivated *fmtC* and *lysC* exhibited reduced lysyl-PG levels and increased susceptibility to cAMPs and certain antibiotics. Specifically, the *fmtC* mutant (HN001) displayed a drop in lysyl-PG content from 3.4% to 0.2%, while the *lysC* mutant (HN002) showed a reduction from 3.4% to 1.3%. Both mutants demonstrated heightened sensitivity to cAMPs, such as β-defensins and CAP18, and antibiotics, such as gentamicin, vancomycin, and moenomycin, highlighting the intricate link between the membrane charge and antibiotic resistance [41]. Furthermore, Kilelee et al. proposed that lysyl-PG primarily affects membrane defect formation. This conclusion was based on binding affinity and dye leakage assays, which revealed that, at 30 mol% concentrations of lysyl-PG, the binding of the +8-charged 6 W-RP-1 peptide remained unaffected. However, dye leakage was almost entirely eliminated, highlighting the role of lysyl-PG in maintaining membrane integrity [19].

Next, Andrä et al. demonstrated that lysyl-PG exerts a protective effect on membranes. In model lipid systems containing only PG, the peptide NK-2 induced significant membrane alterations. However, in lysyl-PG-containing membranes, the ability of NK-2 to form alterations was considerably reduced. Fourier-transform infrared spectroscopy revealed that NK-2 increased the acyl chain order in lysyl-PG-containing membranes, suggesting a rigidifying effect, whereas in the PG-only membrane, it caused a fluidizing effect. Förster resonance energy transfer studies further showed that NK-2 preferentially bound to anionic PG, leading to the formation of PG-enriched domains. In contrast, its interaction with lysyl-PG was less effective, resulting in diminished membrane permeabilization. These findings suggest that lysyl-PG modifies the physical properties of the membrane, thereby reducing its susceptibility to peptide-induced damage [106].

Similarly, studies on daptomycin resistance have shown that lysyl-PG affects the interaction of antimicrobial peptides with bacterial membranes. A prime example of this is the finding that incorporating 20% lysyl-PG into vesicles significantly reduced daptomycin binding affinity, primarily due to a decrease in the electrostatic contribution to the Gibbs free energy of binding. Specifically, the dissociation constant (Kd) for daptomycin increased from 9.4 µM (in the absence of lysyl-PG) to 43 µM with 20% lysyl-PG, indicating a substantial drop in binding affinity. This shift in Kd corresponded to a reduced negative electrostatic contribution to ΔG of approximately −1 kcal/mol, attributed to the inclusion of lysyl-PG. These results demonstrate how lysyl-PG alters the membrane charge distribution, weakening daptomycin interaction with the lipid bilayer [107]. In 2022, further research found that the cationic charge of lysyl-PG did not significantly repel daptomycin or affect membrane fluidity in model systems with lysyl-PG concentrations from 0 to 25%, which was attributed to daptomycin resistance. However, this study revealed that while daptomycin binds less strongly to lysyl-PG than to PG, lysyl-PG promotes increased oligomer formation of daptomycin. This structural change in oligomers may contribute to resistance, suggesting that lysyl-PG affects daptomycin efficacy by altering its oligomeric structure. Since the precise oligomeric configuration is critical for antibacterial activity, this mechanism appears to be more significant than direct electrostatic repulsion [109]. Together, these findings illustrate that while lysyl-PG affects daptomycin binding through electrostatic modifications at lower ratios, it likely contributes to resistance at higher ratios by modifying the structural assembly of daptomycin oligomers. Together, these studies demonstrate that lysyl-PG impacts daptomycin resistance through multiple mechanisms. At lower ratios, it reduces binding affinity via electrostatic modifications, while at higher ratios, it likely promotes resistance by altering daptomycin oligomeric assembly.

In a similar context, Rehal et al. investigated the interaction between lysyl-PG and the antimicrobial peptide magainin 2 F5W. Their study found that lysyl-PG significantly altered the peptide–lipid interaction, particularly when the proportion of lysyl-PG in the membrane exceeded 50% (as observed in extracts at pH 5.5), and the magnitude of the peptide–lipid interaction was notably reduced [37]. Additionally, they found that environmental pH played a critical role in modulating these interactions. At pH 7.4, the model membranes were susceptible to disruption by magainin 2 F5W, indicating that the peptide could effectively partition into the lipid bilayer. In contrast, at pH 5.5, the increased proportion (greater than 50%) of the lysyl-PG analog, named 1,2-O-dipalmitoyl-3-aza-dehydroxy lysylphosphatidylglycerol (DP3adLPG), in the membrane inhibited the formation of the α-helix structure by the peptide. This structural inhibition highlighted how a strong protective effect of lysyl-PG can modulate membrane susceptibility to antimicrobial peptides through both compositional and pH-dependent mechanisms [16].

**Figure 3:**
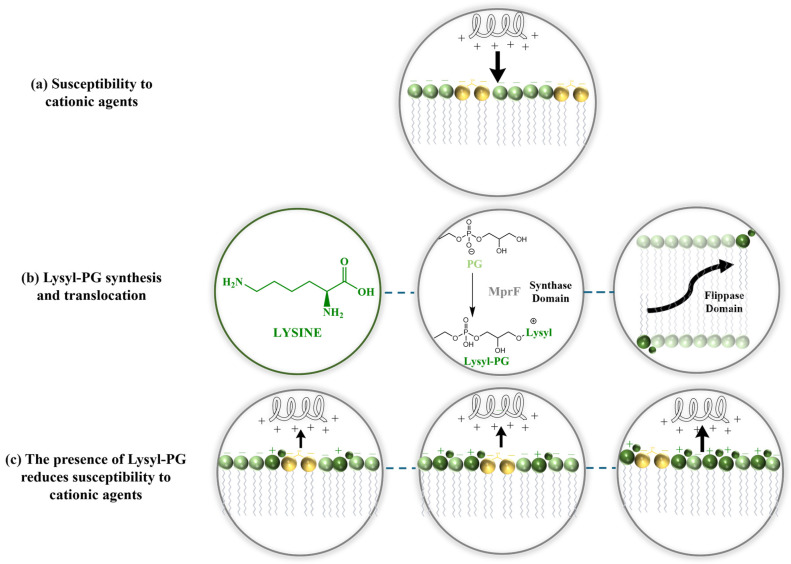
Representative mechanisms for reducing the net negative charge of the bacterial membrane. (**a**) In the absence of lysyl-PG, the bacterial membrane carries a net negative charge, making it highly susceptible to cAMPs. (**b**) The synthesis and translocation of lysyl-PG to the outer leaflet, mediated by the MprF enzyme, reduce the net negative charge of the bacterial membrane. MprF synthesizes lysyl-PG by adding lysine to PG and subsequently translocating it to the outer leaflet. This modification reduces the membrane’s susceptibility to cAMPs, transitioning from the susceptible state shown in panel (**a**) to the resistant state in panel (**c**). (**c**) As the concentration of lysyl-PG increases, binding interactions with antimicrobial cationic agents are diminished, leading to a resistance mechanism.

Further supporting this, research in 2021 revealed that the increased presence of DP3adLPG significantly reduced the binding affinity of magainin 2 F5W. Higher proportions of PG enhanced the peptide α-helical content, suggesting that the anionic nature of 1,2-Dihexadecanoyl-sn-glycero-3-phospho-(1′-rac-glycerol) ammonium salt (DPPG) enhances this interaction. Conversely, as the proportion of DP3adLPG increased, particularly in mixtures approaching equimolar ratios with PG, the peptide helical content decreased, reflecting a reduced binding affinity. The altered charged profile of DP3adLPG likely disrupts electrostatic interactions, impairing the peptide abilities to disrupt the membrane. Additionally, the interaction between PG and DP3adLPG can lead to the formation of ionic pairs, where opposing charges attract, resulting in the formation of lipid complexes. These structures reduce the electrostatic repulsion among lipid headgroups, facilitating the formation of more stable lamellar phases. Such phases influence the membrane’s organization, thickness, packing, and thermotropic properties, further modifying its biophysical characteristics [108]. These findings underscore the critical role of lysyl-PG in *S. aureus* resistance to antimicrobial agents. By shifting the membrane charge profile, lysyl-PG alters the biophysical interactions with antimicrobial peptides and antibiotics, reducing membrane susceptibility. As the concentration of lysyl-PG in the membrane increases, *S. aureus* consistently exhibits reduced susceptibility to different membrane-targeting agents, emphasizing that lysyl-PG plays a critical role in modulating membrane properties, essential those relevant to resistance (Figure 3).

## 8. Environmental Regulation of Lysyl-PG Synthesis

The lipid composition of cell membranes, particularly in *S. aureus*, has been extensively studied, revealing significant variability over time. Several studies consistently showed that the use of enriched or acidic growth media stimulates the production of lysyl-PG, leading to an increased concentration of this lipid in the membrane. Such changes can markedly affect the effectiveness of membrane-targeting antimicrobial agents. Table 3 provides an overview of the methodologies used over the decades to study lipid composition. The table highlights a shift from early techniques, such as chromatographic methods and radioactive labeling, to more advanced high-resolution methods, including nuclear magnetic resonance (NMR) and hydrophilic interaction liquid chromatography.

In 1964, Houtsmuller and Van Deenen conducted groundbreaking experiments on *S. aureus* to investigate the influence of pH on its membrane lipid composition. In their first experiment, the bacteria were cultured in a radioactive glucose-enriched broth, which caused a decrease in pH. When the pH was subsequently adjusted to 7.2 using NaOH, the lysyl-PG content dropped significantly from 38% to 13%, while PG levels increased. In their second experiment, glucose was omitted from the medium, resulting in PG becoming the predominant phospholipid, accounting for 93% of the membrane composition. However, after three additional hours of incubation at a lower pH, PG levels decreased to 35%, accompanied by a corresponding increase in lysyl-PG. Based on these findings, the researchers concluded that pH significantly affects lipid composition, hypothesizing that this positively charged phospholipid plays a critical role in regulating lipid composition. They hypothesized that lysyl-PG, a positively charged phospholipid, helps maintain membrane charge balance in acidic environments [57].

Further experiments conducted in 1965 reinforced these observations. These studies demonstrated that the phospholipid profile of *S. aureus* is highly pH-dependent. At neutral pH 7.0, PG predominated, whereas lysyl-PG constituted over 60% of the membrane lipids at pH levels below 4.8. Importantly, these changes in membrane composition were found to be reversible, highlighting the dynamic adaptability of the bacterial membrane [60]. Next, Hayami et al. continued the research on lipid profiles of *S. aureus* and its derived L-forms, revealing that lysyl-PG is the predominant aminoacyl-PG, with lysine being the only amino acid identified in this lipid fraction. Their findings showed that lysyl-PG was present in both the L-forms and parental strains, though the overall phospholipid composition varied significantly. Notably, the researchers observed an increase in CL levels in L-forms, which is thought to enhance membrane stabilization, compensating for the mechanical protection lost due to the absence of a cell wall [99]. Expanding on this, Koch et al. examined lipid metabolism in *S. aureus*, comparing the incorporation of [^14^C] acetate and [^2–3^H] glycerol to label the lipid fraction and elucidate lipid interactions. Their results showed that lysyl-PG reached a maximum radioactivity of 9.9% and 10.5% mol. During the chase period, lysyl-PG initially displayed an increase in radioactivity, followed by a decrease, which the researchers attributed to the metabolic instability of the lysine residue. These findings suggest that lysyl-PG undergoes rapid turnover, limiting its long-term accumulation within the membrane [110].

Since the discovery of the *mprF* gene in 2001, mutagenesis studies have been conducted to analyze how the resistance profiles of *S. aureus* are affected and to quantify lipid composition in wild-type and mutant strains. These studies revealed a significant decrease in the percentage of lysyl-PG in the mutant strains. Specifically, strain HN001, which carries a genetic insertion in the *fmtC* gene, disrupted its role in lysyl-PG synthesis, and strain HN002, with an insertion in the *lysC* gene, impaired lysine synthesis and exhibited reduced lysyl-PG levels. This reduction increased the negative charge of the bacterial membrane, correlating with heightened susceptibility to certain antibiotics and cAMPs [41]. Building on this research, Parson et al. employed a similar methodological approach to quantify lipid profiles in a mutant strain growth in both non-enriched and glycerol-enriched media. Their findings showed a higher percentage of lysyl-PG in the enriched medium, confirming that the lysyl-PG synthesis is dependent on the availability of PG [111].

Growth and incubation conditions have been shown to significantly influence the lysyl-PG ratio in *S. aureus* membranes. Under oxygen-restricted conditions, Zamudio-Chavez et al. observed a substantial increase in lysyl-PG levels, reporting that oxygen deprivation induces notable changes in both carotenoid and phospholipid composition, including lysyl-PG. These alterations are proposed to play a critical role in maintaining membrane fluidity and electrostatic equilibrium under hypoxic stress by modulating the biophysical properties of the bacterial membrane. This evidence highlights the importance of environmental factors, particularly oxygen availability, in the regulation of phospholipid biosynthesis and membrane adaptability [113].

In a related study, Rehal et al. observed that the lysyl-PG content of five clinical isolates increased in response to environmental pH, which was consistent with previous findings. The degree of response varied among strains, with lysyl-PG accounting for approximately 28% to 55% of the total phospholipids under mildly acidic conditions. At physiological pH, the lysyl-PG content ranged between 28% and 34%. These clinical isolates of *S. aureus* provide critical insights into bacterial behavior in pathological environments and their capacity to develop antibiotic resistance; notably, four of the isolates studied were methicillin-resistant [37]. In 2023, Bimpeh and Hines compared traditional lipid extraction methods, specifically the Bligh and Dyer protocol, with a novel acetic acid-based technique that significantly improved the total yield of lysyl-PG. Their study revealed that in the antibiotic-resistant strain JE2-Dap2, lysyl-PG levels increased by 1.5 to 3 times compared to the non-resistant strain JE2, while PG levels were substantially reduced [112]. This shift in lipid composition highlights a potential link between altered membrane lipid profiles and antibiotic resistance

## 9. The Role of CL and STX in *S. aureus* Membrane Adaptation and Resistance

Bacteria, including *S. aureus*, are well known for their adaptability to external stressors and their capacity to develop resistance to a wide range of antibiotics. *S. aureus* has evolved resistance mechanisms against nearly all clinically used antibiotics, employing different strategies to deactivate these drugs. With the growing use of antimicrobial peptides, research has revealed that *S. aureus* employs lipid synthesis as a key resistance mechanism. Among these lipids, lysyl-PG has emerged as one of the most extensively studied. Over the last decade, it has garnered significant attention for its critical role in neutralizing the negative charge of the bacterial membrane, thereby reducing the binding and effectiveness of cAMPs.

In addition to lysyl-PG, CL and STX play crucial roles in modifying the biophysical properties of bacterial membranes, enhancing their resistance to stress and antimicrobial agents. Molecular dynamic simulations have shown that CL induces several biophysical changes in the membrane that synergistically help maintain membrane integrity. Notably, CL increases the free-energy barrier for transmembrane pore formation and enhances pore kinetic instability, thereby reducing membrane susceptibility to antimicrobial peptides [114]. Further research by Wilson et al. highlighted that variations in CL concentration significantly affect lipid diffusion but have only minor effects on the structural features of the bilayer. They also observed that the position of CL along the bilayer surface correlated with negative curvature deviations, aligning with its role in inducing negative curvature stress in membrane monolayers. This stress is thought to influence membrane dynamics, such as fusion and fission, crucial for the adaptability of mitochondrial membranes [115]. CL, a phospholipid synthesized in *S. aureus* as an adaptive response to external stress, plays a vital role in modulating membrane properties. Increased CL content in liposomes increased the lipid packaging and reduced the lytic activity of the antimicrobial peptides LL-37 and ΔM2 [38]. These findings emphasize the importance of CL in strengthening bacterial membrane defenses against external challenges. On the other hand, STX is a prominent virulence factor in *S. aureus* and a key target for anti-virulence therapies. STX protects the bacteria from oxidative damage, enabling *S. aureus* to survive hostile conditions, such as exposure to the host immune system. Research highlights the potential of targeting STX to develop novel therapies that mitigate the pathogen’s virulence without fostering antibiotic resistance [116].

Molecular dynamics studies have revealed that membrane thickness influences the configurations of STX by altering its relative orientation within the membrane. This orientation prevents the formation of localized clusters, instead creating an interconnected structure throughout the membrane. At a concentration of 15 mol%, STX induces notable changes in membrane properties, including changes in the radial distribution function and an increase in the order parameter of the PG-acyl chains. Additionally, the presence of 15 mol% of STX reduces the membrane diffusion coefficient [117]. STX has also been shown to increase acyl chain order at bacterial growth temperatures and shift the cooperative melting temperature of the membrane, thereby enhancing tolerance to oxidative-stress tolerance and resistance to antimicrobial peptide [47]. Environmental factors, such as oxygen availability, significantly influence the biosynthesis of STX. Under oxygen-restricting conditions, *S. aureus* downregulates carotenoid production, resulting in altered membrane biophysical properties, including increased spacing between lipid headgroups and reduced bilayer core thickness [113]. STX acts as a regulator of *S. aureus* membrane biophysical properties, particularly influencing the mechanical stability of lipid bilayers. This regulation plays a fundamental role in the bacteria homeoviscous adaptation [48]. The main message is that both CL and STX contribute to *S. aureus* resistance by modifying membrane biophysical properties. Their presence enhances membrane stability, reduces susceptibility to pore formation, and increases resistance to antimicrobial peptides. Understanding the role of lipids such as lysyl-PG, CL, and STX in *S. aureus* survival and pathogenicity is critical for developing new strategies to combat antimicrobial resistance. These lipids not only play essential roles in evading host defenses but also contribute to the bacterium’s virulence and persistence in clinical environments.

## 10. Conclusions and Future Perspectives

Antibiotic resistance is one of the most serious threats to public health worldwide. The search for alternative treatments to standard antibiotics is urgent. Studies on *S. aureus* have shown that lysyl-PG acts as a key factor in bacterial resistance mainly by altering the surface charge of the membrane. Synthesized and translocated by the MprF enzyme, lysyl-PG adds a positive charge to the membrane, which reduces the binding affinity of cAMPs, defensins, and antibiotics such as moenomycin, vancomycin, daptomycin, and gentamicin. Lysyl-PG also exerts a protective effect by increasing membrane rigidity, reducing peptide-induced damage, modulating interactions with the membrane, promoting stable lipid domains, and reducing electrostatic repulsion, further enhancing bacterial resistance.

MprF is a potential therapeutic target due to its dual role in lysyl-PG biosynthesis and translocation. Experimental evidence establishes that *mprF*, *fmtC*, and *lysC* are essential for lysyl-PG production, with gene knockout substantially increasing antimicrobial susceptibility. Specific *mprF* mutations drive resistance exclusively through flippase dysfunction while maintaining lysyl-PG synthesis. This bifunctional nature renders MprF inhibition uniquely valuable, as it could concurrently block lipid modification and disrupt the translocation, potentiating the activity of cAMPs.

Building on the insights into the role of lysyl-PG in *S. aureus* resistance, future research could utilize advanced imaging techniques, such as multimodal imaging mass spectrometry (IMS), to further explore the dynamic modifications of bacterial membranes in host infection environments. Perry et al. (2022) demonstrated the effectiveness of multimodal IMS in visualizing *S. aureus* membrane modifications during infection, uncovering heterogeneous distributions of lysyl-PG within bacterial communities in infected tissues from both murine models and human samples. This finding suggests that *S. aureus* exhibits spatially distinct microbial responses to host defenses [118]. This technique could provide valuable insights into the spatial distribution and temporal changes in lysyl-PG in bacterial membranes during infection, offering a more comprehensive understanding of its role in resistance.

Studying the role of lysyl-PG is fundamental for understanding the resistance mechanism in *S. aureus.* However, it is also necessary to investigate alternative lipid modifications in *S. aureus*, explore synergistic antibiotic combinations and cAMP–antibiotic combinations, or develop targeted inhibitors against key enzymatic pathways to design new therapeutic strategies with potent activity against the growing crisis of multidrug-resistant bacteria. Environmental factors, such as pH, amplify the effects of lysyl-PG, emphasizing its critical role in bacterial defense mechanisms. These findings highlight the importance of understanding the complex relationship between lipid composition, membrane biophysical properties, and antimicrobial susceptibility. This knowledge is crucial for developing targeted therapies against *S. aureus* and other antibiotic-resistant pathogens. Ongoing research into lipid-mediated resistance mechanisms is vital for advancing strategies to effectively combat antimicrobial resistance.

## Figures and Tables

**Table 1:** Reported bacterial species with the presence of MprF or homologs producing lysyl-PG.

Bacteria or Species	Type	Strain	Concentration (Lysyl-PG)	Technique	Ref.
*Agrobacterium* *tumefaciens*	Gram-negative	C58	~1%	HPTLC and 2DTLC	[79]
*Anoxibacillus* *rupiensis*	Gram-positive	Is not named	2.8–23.2% (pH 6.5)	HILIC and LC–ESI–MS/MS	[78]
*Bacillus Anthracis*	Gram-positive	Sterne 34F2	+	2D-TLC profiles of ^32^P-labeled phospholipids and MS	[80]
*Bacillus* *licheniformis*	Gram-positive	ATCC 14580	3%	2DTLC	[81]
*Bacillus megaterium*	Gram-positive	MK 10D MK 10D	8–14% 15–16%	Phospholipids were analyzed from extracted ^32^P-labeled lipids located in the chromatogram by autoradiography.	[70] [82]
*Bacillus siamensis*	Gram-positive	PD-A10^T^	+	TLC	[83]
*Bacillus subtilis*	Gram-positive	Marburg	22% (pH: 7.0) 42% (pH: 5.0)	Phospholipids were analyzed from extracted ^32^P-labeled lipids located in the chromatogram by autoradiography.	[84]
		SDB110, YB886 and mutants	8–31%	HPTLC	[85]
		168 and BKE08425	+	TLC and MS/MS	[86]
		168	+	MS/MS	[87]
		168	8.5%	^31^P NMR	[88]
*Bacillus* *thuringiensis*	Gram-positive	ATCC 10792	10%	2DTLC	[81]
*Caulobacter* *acrescentes*	Gram-negative	CB13	10.4–11.4%	Phospholipids were analyzed from extracted ^32^P-labeled lipids located in the chromatogram by autoradiography.	[89]
*Clostridium perfringens*	Gram-positive	Is not named ATCC 3624	+ +	TLC and infrared spectra TLC and MS	[55] [90]
*Cohnella* *boryungensis*	Gram-positive	BR-29^T^	+	2DTLC	[91]
*Enterococcus faecalis*	Gram-positive	10C_I_	20_exp_–35%_stat_	Incorporation of L-[^14^C] lysine into lipids and chromatography on Silicic acid impregnated paper	[71]
		ATCC 9790	+	Autoradiogram of ^32^P-labeled phospholipids	[92]
		OG1RF, Dap21 and Dap22	+	LC-MS/MS	[93]
		OG1RF	0.05–2.97 μM	HILIC and high-resolution MS	[94]
*Lactobacillus* *species*	Gram-positive	*L. casei* *L.arabinosus* 9K *L.plantarum* 9P *L.acidophilus* 9MB *L. lactis* 9T *L.bulgaricus* 9LB *L. fermenti* 9H	27–30% 14% 23% 3% 23% 32% 14%	2DTLC Autoradiogram of ^32^P-labeled phospholipids. The data are expressed as a percentage of total lipid phosphorus radioactivity.	[95]
*Listeria innocua*	Gram-positive	NCTC 11288^T^	8.6%_stat_–12.3%_exp_	2DTLC. Data are the percentage of total lipid phosphorus	[69]
*Listeria* *monocytogenes*	Gram-positive	NCTC 7973	5.4%_exp_	2DTLC. Data are the percentage of total lipid phosphorus.	[69]
		EGD-e	+	2DTLC, ESI-MS, and GC-MS	[96]
		EGD-e	0–54% (30 °C) 1–73% (37 °C)	2DTLC of ^32^P-phospholipids	[66]
*Listeria seeligeri*	Gram-positive	SLCC 3954^T^	2.9%_exp_	2DTLC. Data are the percentage of total lipid phosphorus	[69]
*Listeria welshimeri*	Gram-positive	SLCC 5334^T^	10.8%_exp_	2DTLC. Data are the percentage of total lipid phosphorus	[69]
*Mammaliicoccus* *sciuri*	Gram-positive	SCH89 SCH91 (DMS20352, DMS20345)	+	2DTLC and MS	[63]
*Micrococcus luteus*	Gram-positive	B-P26	+	TLC	[97]
*Mycobacterium* *tuberculosis*	Gram-positive	Rv-03 Rv-80lys Rv-81ami Rv-82med	+	^14^C lysine-labeled lipid and TLC plates were either visualized by autoradiography, MALDI-MS, and ^31^P-NMR	[73]
*Pseudomonas* *aeruginosa*	Gram-negative	NCTC 6750	+	Spectrophotometric techniques	[72]
*Rhizobium tropici*	Gram-negative	CIAT899	0.6–1.2%	^14^C lysine-labeled lipid and TLC	[98]
*Staphylococcus* *aureus*	Gram-positive	Is not named COL, HN001 and HN002	1–70% 0.2–3.4%	Is not named Radiolabeling. Data expressed as percentages of total radioactivity	[57] [41]
*L-form S. aureus*	Gram-positive	Newman and Tazaki	6.1–17.3%	2DTLC on silica gel G plates, ^32^P-labeled or detected by rhodamine G6	[99]
*Staphylococcus* *capitis*	Gram-positive	NCTC 11045	+	2DTLC and MS	[63]
*Staphylococcus cohnii*	Gram-positive	NCTC 11041	+	2DTLC and MS	[63]
*Staphylococcus* *epidermidis*	Gram-positive	NCTC 11047 Skin isolated	+ +_exp_	2DTLC and MS TLC and MS/MS	[63] [100]
*Staphylococcus* *haemolyticus*	Gram-positive	Skin isolated NCTC 11042	+ +_exp_	2DTLC and MS TLC and MS/MS	[63] [100]
*Staphylococcus* *hominis*	Gram-positive	NCTC 11320	+	2DTLC and MS	[63]
*Staphylococcus* *hyicus*	Gram-positive	NCTC 10530	+	2DTLC and MS	[63]
*Staphylococcus* *intermedius*	Gram-positive	NCTC 11048	+	2DTLC and MS	[63]
*Staphylococcus* *saprophyticus*	Gram-positive	SCH94 and SCH95	+	2DTLC and MS	[63]
*Staphylococcus* *simulans*	Gram-positive	NCTC 11046	+	2DTLC and MS	[63]
*Staphylococcus warneri*	Gram-positive	NCTC 11044	+	2DTLC and MS	[63]
*Staphylococcus* *xylosus*	Gram-positive	NCTC 11043	+	2DTLC and MS	[63]
*Streptococcus* *agalactiae*	Gram-positive	COH1A909	+	NPLC-ESI/MS	[101]
*Streptococcus* *thermophilus*	Gram-positive	ATCC 19258	10%	2DTLC	[81]
*Vagococcus fluvialis*	Gram-positive	NCDO 2497	10.1%_stat_–21.2%_exp_	HPLC and TLC. Abundance (mol%) at phase	[102]

(+) denotes the presence of lysyl-PG. The quantity and specific amounts are provided as far as they are known. (exp) refers to the exponential growth phase, while (stat) refers to the stationary growth phase.

**Table 2:** Summary of research works that analyze the interaction of antimicrobial agents with *S. aureus* membrane models containing lysyl-PG or synthetic analogs.

Lipid Model	Strain	Composition	Antimicrobial Agent	Sequence	Result	Charge	Ref.
Liposomes	Wild-type: Newman and Sa113	Bacterial extracts from wild-type strains have lysyl-PG concentrations up to 38%	Defensin HNP-1 *	ACYCRIPACIAGERRYGTCIYQGRLWAFCC (Cys2-Cys30,Cys4-Cys19,Cys9-Cys29)	The mutant strains were significantly more susceptible to a broad range of antimicrobial agents, while the neutral gramicidin D exhibited equal activity against both wild-type and mutant strains. Enhanced binding of mutant cells to a tachyplesin 1 and gallidermin further demonstrated that lysyl-PG reduced the attraction and binding of antimicrobial agents.	+3	[15]
			Protegrins 3	RGGGLCYCRRRFCVCVGR-NH_2_ (Cys6-Cys15,Cys8-Cys13)		+6	
			Protegrins 5	RGGRLCYCRPRFCVCVGR-NH_2_ (Cys6-Cys15,Cys8-Cys13)		+5	
			Tachyplesin 1	KWCFRVCYRGICYRRCR-NH_2_ (Cys3-Cys16,Cys7-Cys12)		+6	
			Gallidermin	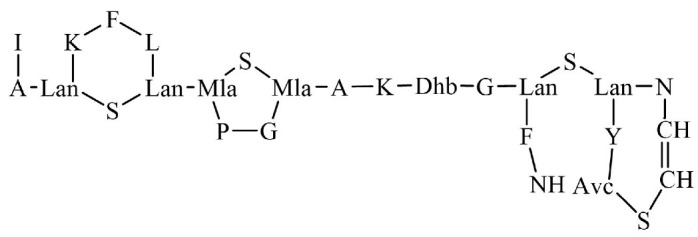		-	
	Mutants: pBR473 and pRBmprF	Mutant strains have 0% lysyl-PG	Nisin	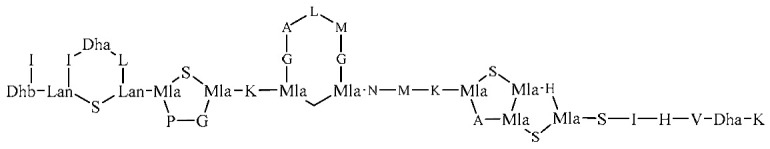		-	
			Magainin II	GIGKFLHAAKKFAKAFVAEIMNS-NH_2_		+4	
			Melittin	GIGAVLKVLTTGLPALISWIKRKRQQ-NH_2_		+5	
			Gramicidin S	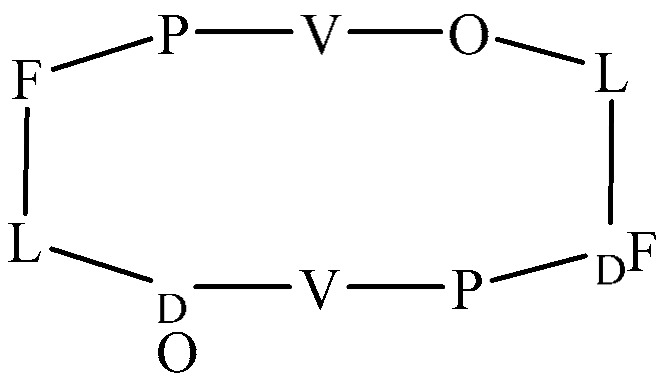		-	
			Gramicidin D	HCO-VGA_D_LA_D_VW_D_LW_D_LW-NHCH_2_CH_2_OH		0	
Vesicles	Wild-type: SA113 mutants *mprF*^−^ *dltA*^−^	Bacterial extracts from wild-type and *dltA* strains contain lysyl-PG, while the *mprF^−^* mutant does not produce lysyl-PG	Human Group IIA Phospholipase A_2_		Mutations affecting the charge properties of the bacterial envelope had a significant impact on PLA2 activity. For instance, *dltA^−^* deficient mutants, unable to modify teichoic acids with D-alanine, exhibited a sensitivity to PLA2 that was 30 to 100 times greater than that of the parental strain. In contrast, mprF-deficient bacteria, which lacked lysyl-phosphatidylglycerol synthesis, displayed only a modest increase in susceptibility to PLA2, with a fold change of no more than three (≤3-fold) compared to the wild-type *S. aureus* strain. These findings highlight the distinct roles of *dltA^−^* and *mprF^−^* in modulating bacterial envelope properties and their differential effects on resistance to PLA2.	Ranging from +12 to +17	[105]
Is not named	Wild-type: COL Mutants: HN001 HN002	Bacterial extract lysyl-PG:PG:GL:CL 3.4:90:5.6:1 0.2:89.7:7.3:2.8 1.3:89.0:7.0:2.7	Moenomycin	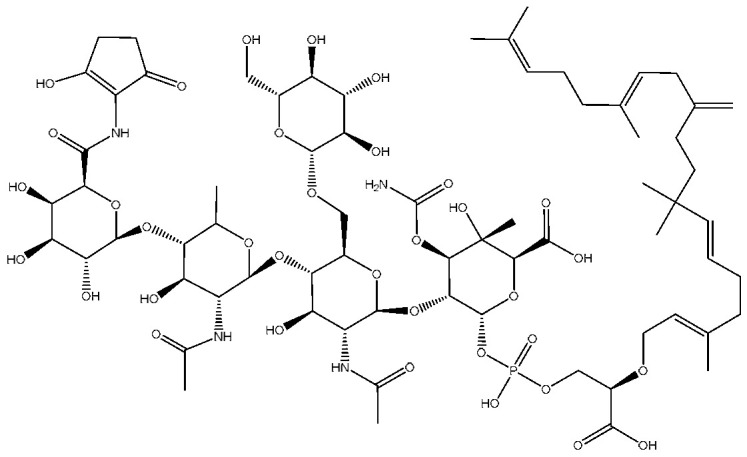	The mutants showed slightly decreased susceptibility to moenomycin and vancomycin, they were more vulnerable to positively charged antimicrobial agents (e.g., α-defensins and CAP18) due to the increased negative charge of their membranes.	−1	[41]
			Vancomycin	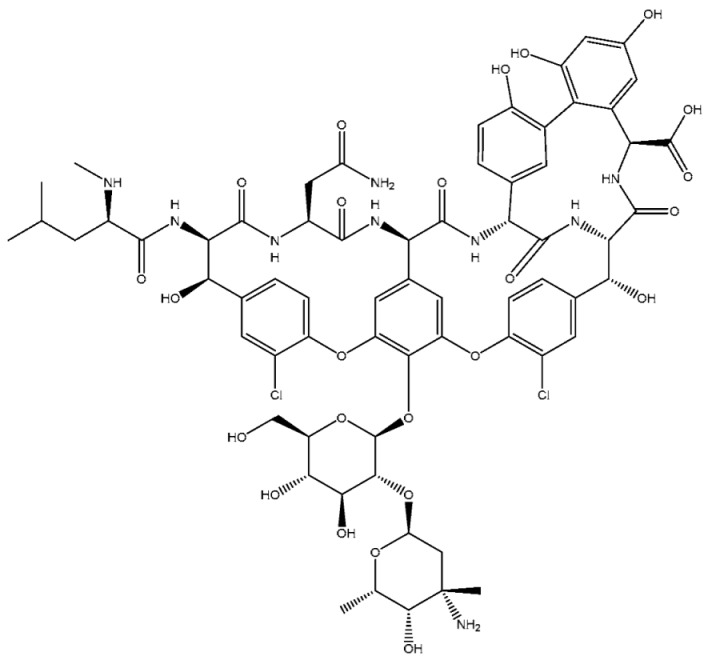		+1	
			hBD3	IINTLQKYYCRVRGGRCAVLSCLPKEEQIGKCSTRGRKCCRRKK		+11	
			CAP18 (Precursor of LL-37)	LLGDFFRKSKEKIGKEFKRIVQRIKDFLRNLVPR		+7	
Synthetic LUVs	-	POPG:POPC:Lysyl-DOPG 70:30:0 50:30:20 40:30:30 30:30:40	6W-RP-1	ALYKKWKKKLLKSLKRLG	The presence of lysyl-PG significantly inhibited dye leakage induced by the antimicrobial peptide 6W-RP-1. Additionally, the authors suggested that the cationic peptide preferentially interacted with anionic PG, leading to the formation of PG-enriched lipid domains and the exclusion of cationic lysyl-PG.	+8	[19]
LUVs and planar bilayers	Clinical isolate wild-type: Sa113 (ATCC 35556) Mutants: SA113-derived (Δ*mprF*), and Δ*mprF*-derived (Δ*mprFpRBsyn*)	PE:DOPG:Lysyl-DOPG 50:0:50 0:100:0 0:50:50 50:25:25	NK-2	KILRGVCKKIMRTFLRRISKDILTGKK-NH_2_	The presence of lysyl-PG in the membrane significantly reduced the effectiveness of the cAMPs. The mutant strains lacking lysyl-PG were more susceptible to the tested peptides compared to the wild-type strain. NK-2 increased the acyl chain order in lysyl-PG-containing membranes, indicating a rigidifying effect, while it had a fluidizing effect in PG-only membranes.	+10	[106]
			C7A	KILRGVAKKIMRTFLRRISKDILTGKK-NH_2_		+10	
			C7S/NK27	KILRGVSKKIMRTFLRRISKDILTGKK-NH_2_		+10	
			NK23a	KISKKIMRTFLRRISKDILTGKK-NH_2_		+9	
			NK23b	KILRGVSKKIMRRISKDILTGKK-NH_2_		+9	
			NK23c	KILRGVSKKIMRTFLRRILTGKK-NH_2_		+10	
			NK11	KISKRILTGKK-NH_2_		+6	
			Melittin	GIGAVLKVLTTGLPALISWIKRKRQQ-NH_2_		+5	
			Ar-1	RWCVYAYVRVRGVLVRYRRCW-OH		+6	
			C/S-Ar-1,	RWSVYAYVRVRGVLVRYRRSW-OH		+6	
			R/K-Ar-1	KWCVYAYVKVKGVLVKYKKCW-OH		+6	
Synthetic LUVs	-	POPC:POPG:lysyl-PG 70:30:0 70:10:20	Daptomycin	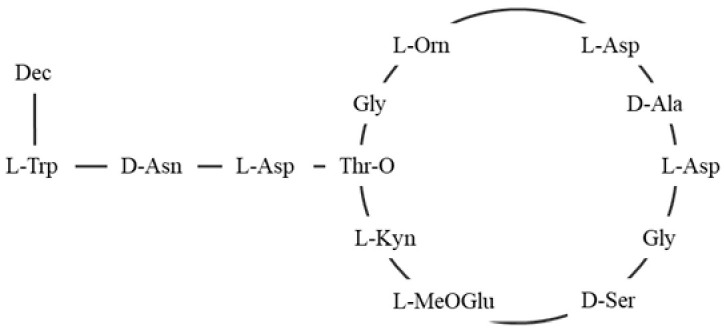	The presence of lysyl-PG in lipid vesicles moderately reduced the binding affinity of daptomycin. The reduction in daptomycin binding affinity was linked to a decrease in the electrostatic contribution to the Gibbs free energy of binding, caused by the cationic nature of lysyl-PG. This indicated that the interaction between daptomycin and the lipid bilayer was affected by the overall charge distribution in the membrane.	−2	[107]
Lipid extract monolayer	476	PG:lysyl-PG:CL 43:51:6	Magainin 2 F5W	GIGKWLHSAKKFGKAFVGEIMNS	Lysyl-PG biosynthesis in *S. aureus* increased significantly under mildly acidic conditions. This modification helped to neutralize the membrane’s surface charge and increased membrane rigidity, enhancing stability under stress. The ionization of the headgroup amine of lysyl-PG at acidic pH reduced interactions between antimicrobial peptides and the membrane, providing protection against lysis.	+3	[37]
		62:33:4					
	MRSA G32	40:52:8					
		67:28:5					
	MRSA G33	66:28:6					
		58:34:9					
	MRSA H64	50:44:6					
		64:33:4					
	MRSA H66	40:55:5					
		61:34:5					
Synthetic Monolayer and vesicles	-	Molar ratios: DPPG:DP3adLPG:TMCL 67:28:5 41:51:8 d_62_DPPG/d_62_DP3adLPG 70:30 45:55	Magainin 2 F5W	GIGKWLHSAKKFGKAFVGEIMNS	The presence of lysyl-PG in the lipid bilayer altered the membrane’s charge characteristics, making it predominantly zwitterionic at neutral pH and cationic in mildly acidic conditions, which reduced the peptide’s ability to adopt its active α-helical conformation necessary for membrane disruption.	+3	[16]
Synthetic Liposomes	-	DPPG:DP3adLPG 70:30 60:40 55:45 40:60 30:70 DPPC:DPPG 75:25	Magainin 2 F5W	GIGKWLHSAKKFGKAFVGEIMNS	Magainin 2 F5W showed a higher binding affinity and α-helical content in DPPG-rich environments, indicating that anionic lipids enhanced its interaction. The formation of distinct lamellar and non-lamellar phases in these mixtures further altered membrane properties, influencing the peptide’s mechanism.	+3	[108]
Synthetic LUVs	-	DMPC:Lysyl-DMPG 75:25 DMPC:DMPG 75:25 DMPG:DMPC:Lysyl-DMPC 12.5:0:87.5 12.5:6.25:81.25 12.5:12.5:75 12.5:25:62.5 12.5:18.75: 68.75 0:25:75 25:25:50 25: 0:75	Daptomycin	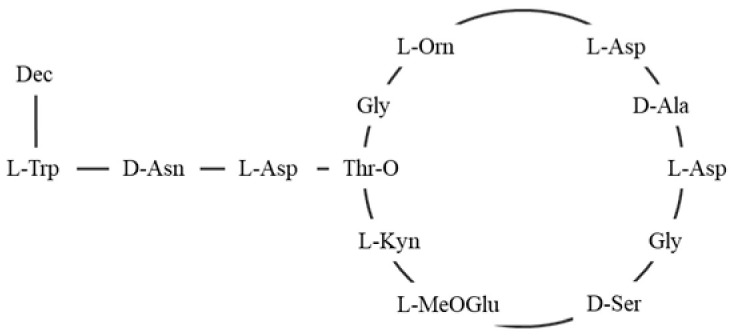	The findings suggested that lysyl-PG could affect the oligomerization of daptomycin, potentially altering its antibacterial activity, indicating that while lysyl-PG could mask the binding sites for daptomycin, the overall impact on membrane fluidity and the structural conformation of daptomycin remained marginal.	−2	[109]

* Although human defensin HNP-1 is listed, the antibacterial assay utilized a mixture of HNP-1, HNP-2, and HNP-3. These defensins differ only in their first amino acid but share similar antimicrobial properties.

**Table 3:** Publications based on the quantification of *S. aureus* membrane lipids.

Strain	Conditions	Lipid Composition			Technique	Results and Notes	Ref.
		PG	Lysyl-PG	CL			
Is not named	I: pH 4.8 F: pH 7.2 Glucose + 16 h, 37 °C	~50%	~38%	~12%	The percentage of phospholipids was measured by analysis of phospholipid composition in bacterial cultures and TLC. Data are expressed as a percentage of total lipids. The percentages of phospholipids were extracted using PlotDigitizer, as the scientific article does not directly provide the values.	The amount of lysyl-PG increased significantly when glucose was present, while the relative amount of PG decreased.	[57]
	I: pH 7.2 F: pH 7.2 Glucose + 16 h 37 °C The pH was adjusted to 7.2 and incubated again for 3 h	~79%	~13%	~9%			
	I: pH 7.2 F: pH 7.4 Glucose – 16 h, 37 °C	~93%	~4%	~3%			
	I: pH 7.2 F: pH 4.8 Glucose − Incubated for more 3 h	~35%	~58%	~6%			
	pH range: 7.2–6.2 Glucose + 12 h	~63%	~22%	~17%			
	pH range: 5.2–4.8 Glucose -	~21%	~70%	~11%			
PS 187 (NCTC 9754)	pH: 4.7 A	30.5%	67.5%	-	Radiolabeled lipids and chromatography. Cells harvested at pH 4.7 (A) and 7.2 (B) were extracted according to the procedure of Bligh and Dyer. Data expressed as a percentage of total lipids and the values are the means of six experiments and are expressed as mg lipid per g of lyophilized cells.	The phospholipid composition varied significantly with changes in the pH of the culture medium. Specifically, CL was observed to accumulate at low pH values, while the percentages of PG and lysyl-PG changed ostensibly with pH adjustments.	[60]
	pH: 6.3 A	44.0%	54.0%	-			
	pH: 7.0 A	72.5%	25.5%	-			
	pH: 4.7 B	89.0%	9.0%	-			
	pH: 6.3 B	90.0%	8.0%	-			
	pH: 7.0 B	94.0%	4.0%	-			
	pH: 7.2	19.3 mg	3.8 mg	0.5 mg			
	pH: 4.8	3.2 mg	5.8 mg	3.0 mg			
Newman	L-forms were grown in brain heart infusion broth (Difco) supplemented with 5% NaCl and 10% horse serum on a reciprocal shaker at 37 °C.	43.1	30.1	22.5	Two-dimensional chromatography on silica gel G plates. Data expressed as percentages of phosphorus in total phospholipids.	The analysis revealed that lysyl-PG was identified as the primary aminoacyl-PG in *S. aureus*, with lysine being the only amino acid detected in this lipid fraction. The L-forms showed a decrease in lysyl-PG and PG content, and a significant increase in CL content, which exceeded 50% of the total phospholipid phosphorus, compared to less than 25% in the parental strains.	[99]
Newman Lf		26.1	17.3	53.9			
Tazaki		43.1	35.1	17.9			
Tazaki Lf		11.8	6.1	78.6			
DSM 346	This involved the labeling of logarithmically growing bacteria with 44 µM [^14^C] acetate.	50.4%	9.9%	1.1%	Data expressed as %mol. The mole percentage of these lipids was determined under different labeling conditions, revealing their relative abundance in the bacterial membrane.	The lipid composition in *S. aureus* showed that PG, lysyl-PG, and bisphosphatidylglycerol (CL) were among the key components. The type of radioactive labeling did not affect the quantification of lysyl-PG.	[110]
	This involved the labeling of the bacteria with 5 mM [^2–3^H] glycerol.	54.5%	10.5%	0.7%			
COL (wild-type)	*S. aureus* strains were grown in trypticase soy broth.	90%	3.4%	1.0%	Radiolabeling with [^2–3^H] glycerol. Data expressed as percentages of total radioactivity.	The *fmtC* gene played a crucial role in the synthesis of lysyl-PG. When this gene is disrupted, as observed in mutant HN001, the production of lysyl-PG is significantly reduced. Similarly, the *lysC* gene is involved in the biosynthesis of lysine, which serves as a precursor for lysyl-PG. A mutation in this gene, as seen in mutant HN002, also leads to decreased levels of lysine available for lysyl-PG synthesis, further contributing to the reduction in lysyl-PG in the membrane.	[41]
HN001 mutant (*fmtC*)		89.7%	0.2%	2.8			
HN002 mutant (*lysC:*)		89.0%	1.3%	2.7			
PDJ28 (Δ*gpsA*)	With glycerol	55.0	23.2	<1	TLC, lipid mass spectrometry, and radiolabeling. Data expressed as percentages of total ^14^C-label.	Removal of glycerol from the growth medium led to the rapid cessation of phospholipid synthesis.	[111]
	Without glycerol	28.4	18.4	12.5			
476	pH: 5.5	43%	51%	6%	^31^P NMR. Data expressed as a percentage of total phospholipid content.	Mildly acidic conditions significantly stimulate the biosynthesis of lysyl-PG in *S. aureus*. This study found that under these conditions, the proportion of lysyl-PG in the total phospholipid content increased markedly, with some strains showing levels as high as 50% of total phospholipids.	[37]
	pH: 7.4	62%	33%	4%			
MRSA G32	pH: 5.5	40%	52%	8%			
	pH: 7.4	67%	28%	5%			
MRSA G33	pH: 5.5	66%	28%	6%			
	pH: 7.4	58%	34%	9%			
MRSA H64	pH: 5.5	50%	44%	6%			
	pH: 7.4	64%	33%	4%			
MRSA H66	pH: 5.5	40%	55%	5%			
	pH: 7.4	61%	34%	5%			
JE2 (NR-4653) and JE2-Dap2	Bligh and Dyer	13.07 µM	0.10 µM	-	HILIC and MS. Data expressed in µM.	This study found that the addition of 0.5% (*v*/*v*) acetic acid during the extraction process led to a twofold increase in the total yields of lysyl-PG across all species analyzed.	[112]
	Methanol/acetonitrile/water, method developed for the recovery of lipids from bacteria	25.48 µM	0.18 µM	-			

## Data Availability

Not applicable.

## Abbreviations

The following abbreviations are used in this manuscript:

2D TLC	Two-Dimensional Thin-Layer Chromatography
^31^P NMR	Phosphorus-31 Nuclear Magnetic Resonance
aaPGSs	aminoacylphosphatidylglycerol synthases
cAMPs	Cationic Antimicrobial Peptides
CL	Cardiolipin
d62DP3adLPG	d62 1,2 O dipalmitoyl 3 aza dehydroxy lysyl phosphatidylglycerol, trifluoroacetate salt
d62DPPG	d62 1,2 O dipalmitoyl sn glycero 3 phospho (1′ rac glycerol), triethyl ammonium salt
DMPG	1,2-Dimyristoyl-sn-glycero-3-phospho-rac-(1-glycerol) sodium salt
DMPC	1,2-Dimyristoyl-sn-glycero-3-phosphocholine
DOPG	1,2-Dioleoyl-sn-glycero-3-phosphoglycerol
DP3adLPG	Dipalmitoyl-3-aza-dehydroxy-lysylphosphatidylglycerol
DPPC	1,2-Dipalmitoyl-rac-glycero-3-phosphocholine
DPPG	1,2-Dihexadecanoyl-sn-glycero-3-phospho-(1′-rac-glycerol) ammonium salt
GL	Glycolipid
GLASS	Global Antimicrobial Resistance and Use Surveillance System
HILIC	Hydrophilic Interaction Liquid Chromatography
HPLC	High-Performance Liquid Chromatography
HPTLC	High-Performance Thin-Layer Chromatography
LC/ESI–MS/MS	Liquid Chromatography–Electrospray Ionization Tandem Mass Spectrometry
LC/MS	Liquid Chromatography–Mass Spectrometry
LC-MS/MS	Liquid Chromatography–Tandem Mass Spectrometry
LUVs	Large Unilamellar Vesicles
lysyl-PG	lysyl-phosphatidylglycerol
lysyl-DMPG	1,2-Dimyristoyl-sn-glycero-3-[phospho-1-(3-lysyl(1-glycerol))]
lysyl-DOPG	1,2-Dioleoyl-sn-glycero-3-[phospho-rac-(3-lysyl(1-glycerol))]
MprF	Multiple peptide resistance factor
MALDI-MS	Matrix-Assisted Laser Desorption/Ionization Mass Spectrometry
MS	Mass Spectrometry
NPLC-ESI/MS	Normal Phase Liquid Chromatography Coupled with Electrospray Ionization Mass Spectrometry
PE	Phosphatidylethanolamine
PG	Phosphatidylglycerol
POPC	1-Palmitoyl-2-oleoyl-sn-glycero-3-phosphocholine
POPG	1-Palmitoyl-2-oleoyl-sn-glycero-3-(phospho-rac-(1-glycerol))
*S. aureus*	*Staphylococcus aureus*
STX	Staphyloxanthin
Tandem MS	MS/MS
TLC	Thin-Layer Chromatography
TMCL	1,1′,2,2′-Tetramyristoyl cardiolipin
